# Predicting candidate genes for human deafness disorders: a bioinformatics approach

**DOI:** 10.1186/1471-2164-7-180

**Published:** 2006-07-19

**Authors:** Rami Alsaber, Christopher J Tabone, Raj P Kandpal

**Affiliations:** 1 Department of Biological Sciences, Fordham University Bronx, NY 10458, USA

## Abstract

**Background:**

There are more than 50 genes for autosomal dominant and autosomal recessive nonsyndromic hereditary deafness that are yet to be cloned. The human genome sequence and expression profiles of transcripts in the inner ear have aided positional cloning approaches. The knowledge of protein interactions offers additional advantages in selecting candidate genes within a mapped region.

**Results:**

We have employed a bioinformatic approach to assemble the genes encoded by genomic regions that harbor various deafness loci. The genes were then *in silico *analyzed for their candidacy by expression pattern and ability to interact with other proteins. Such analyses have narrowed a list of 2400 genes from suspected regions of the genome to a manageable number of about 140 for further analysis.

**Conclusion:**

We have established a list of strong candidate genes encoded by the regions linked to various nonsyndromic hereditary hearing loss phenotypes by using a novel bioinformatic approach. The candidates presented here provide a starting point for mutational analysis in well-characterized families along with genetic linkage to refine the loci. The advantages and shortcomings of this bioinformatic approach are discussed.

## Background

Hearing loss, acquired or genetic, is a major worldwide public health concern. Numerous genes have been linked to hearing disorders [1]. These disorders may be syndromic or nonsyndromic; conductive, sensorineural, or mixed; and prelingual or postlingual [2]. The various genetic forms of hearing loss are distinguished based on otologic, audiologic and physical examination combined with linkage analysis. Some representative deafness genes that have been identified include the Alport syndrome (*COL4A3, COL4A4 or COL4A5 genes*), branchio-oto-renal syndrome (*EYA1 *gene), Mohr-Tranebjaerg syndrome (*TIMM8A *gene), Pendred syndrome (*SLC26A4 *gene), Jervell and Lange-Nielsen Syndrome (*KVLQT1 and KCNE1 genes*), Usher syndrome with its several types, Norrie disease (*NDP gene*), DFNB1 (*GJB2 *gene), DFN3 (*POU3F4 *gene), DFNB4 (*SLC26A4 *gene), DFNA6/14 (*WFS1 *gene), and several others [3,4]. The mutational analysis of genes such as *GJB2 *(encoding the protein connexin 26) and *GJB6 *(encoding the protein connexin 30) [3,5,6] has aided diagnosis and geneticcounselling.

Syndromic hearing loss is associated with a variety of other clinical findings and is relatively less prevalent. In contrast, nonsyndromic hearing loss accounts for more than 70% of deafness cases and involves autosomal as well as X or Y -linked deafness phenotypes [7]. The molecular causes of nearly all nonsyndromic hearing loss are associated with inner ear structural damage, and changes in both the inner and the middle ear [8]. Mutations in genes such as the *ACTG1*, *COCH*, *COL11A2*, *DFNA5*, *EYA4*, *GJB2*, *GJB6*, *KCNQ4*, *MYO6*, *MYO7A*, *TECTA*, *TMC1*, and *WFS1*, as well as altered expression of genes such as *GJB3 *and *MYO1A *have been associated with the autosomal dominant types that are generally progressive and involve changes in inner ear [9-11]. The autosomal recessive phenotypes are associated with mutations in genes such as the *CDH23*, *CLDN14*, *ESPN*, *GJB2*, *GJB6*, *MYO15A*, *MYO6*, *MYO7A*, *OTOF*, *PCDH15*, *SLC26A4*, *STRC*, *TECTA*, *TMC1*, *TMIE*, *TMPRSS3*, and *USH1C*, as well as altered expression of *GJB3 *[8].

The map locations of a large number of nonsyndromic autosomal recessive deafness phenotypes are known, but the specific genes responsible for all these phenotypes have not been identified [4]. The cloning of genes involved in such phenotypes requires refinement of the suspected genomic interval to as short a region as possible by linkage analysis. However, it is not always possible to map a gene within an interval that is amenable for mutation analysis. The mutation analysis of all genes encoded by a large genomic interval is extremely labor-intensive. We describe here a bioinformatic approach that can reduce the candidate genes to a manageable number for mutation analysis. Initially, all the genes from a particular locus are cross-referenced to the databases of expressed mouse inner ear genes and the expressed human cochlear genes. The alternative procedure included a search for interacting proteins for the gene products mapping to the candidate region. As presented here, this approach has led to a set of specific candidate genes.

## Results and discussion

**Figure 1:**
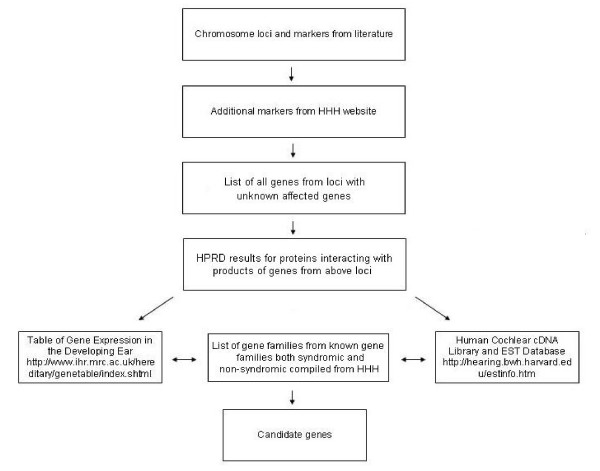
Schematic flow for information processing to predict candidate genes. The rectangles contain tasks that were processed in the sequence as indicated by arrows.

The locations of 23 autosomal dominant and 27 autosomal recessive nonsyndromic deafness phenotypes mapped to several chromosomes downloaded from hereditary hearing loss homepage are shown in Tables 1 and 2[4]. Additional loci for nonsyndromic conditions are mapped to chromosomes 1, 8, X and Y [4]. The hereditary hearing loss homepage is updated on a regular basis. The marker boundaries of these locations encompass between 1.4 and 18.6 million basepairs (Mbp) for various loci. To generate a set of candidate genes for the listed loci, a strategy schematically represented in Figure 1 was followed. The determination of coding sequences and/or genes in a genomic region was made by Unigene [12]. However, the genes encoded in a large genomic interval are too many to be characterized by mutational analysis in a gene-by-gene approach. Therefore, we used the human cochlea and mouse inner ear expression databases [13,14] to eliminate from the candidate list certain genes that were not expressed in these organs. Such *in silico *expression analysis relies on the assumption that the expression databases are comprehensive. However, the characterization of all transcripts expressed in the ear is far from complete. We, therefore, introduced another step in our candidate gene strategy by taking advantage of the human protein reference database (HPRD) and generated a list of interacting genes for every gene mapping to candidate deafness loci [15]. The rationale for protein interaction is as follows. If a gene encoded in the candidate region interacts with a gene that is either involved in inner ear development/function, or a protein shows interaction with more than one candidate genes mapping to different loci, then such a gene is likely to be involved in the phenotype in question. The interaction pattern of the gene products from Usher syndrome is a good example to illustrate this point. The known gene products for several Usher syndrome loci are known to form interactions *in vivo *[16]. The mutation of each one of these genes affects protein interactions and influences Usher type 1 phenotype [17]. The five forms of Usher syndrome have defects in myosin VIIA, harmonin, cadherin 23, protocadherin 15, and a putative scaffolding protein sans. Harmonin binds sans, and it also binds myosin VIIA and protocadherin 15 [34]. The role of cadherins in mediating cell-cell interaction is well-characterized. Furthermore, interactions of harmonin (*USH1C*) with *USH2A*, *USH2C *and *USH2B *are mediated by PDZ domains [35,36]. In retrospect, if the interacting protein strategy had been used to select candidate genes for Usher syndrome subtypes, it is likely that several genes could have been eliminated from consideration. Therefore, it is reasonable to assume that physical interactions will exist between proteins that are involved in inner ear developmental pathway or inner ear signal transduction pathways, and mutations in any one protein of the pathway is likely to give the same altered phenotype. If proteins of interacting networks can be identified or predicted, then such genes are natural candidates for a given phenotype. The above hypothesis is the underlying rationale for incorporating interacting proteins as a criterion for selecting candidate genes presented in this paper. Briefly, the strategy is as follows. First, assemble the genes encoded in all candidate intervals, list the proteins that interact with genes in the candidate region, and then search for candidates on different loci that interact with a common protein. Such a criterion will fulfil the rationale of putative involvement of proteins at two different loci involved in a common biological process, and by association the respective genes mapping to two different loci will be considered as candidates.

The application of candidate gene isolation is demonstrated for the autosomal dominant condition DFNA27. The gene is mapped to the genomic interval 4q12 spanning 15 Mbp [4]. This region codes for 36 known and 30 hypothetical proteins (Table 3) [18]. The comparison of these genes to expression databases reduced the list to 10 genes from the human cochlear database and three found in the mouse inner ear (Table 4) [13,14,19]. The possibility remained for the elimination of a stronger candidate just on the basis that it did not score a hit in expression databases. To avoid such an error, we have assembled lists of interacting proteins by using the human protein reference database (HPRD) [15] for every gene identified by GeneRetriever^® ^from the candidate region. If an interactor of a gene in the candidate interval is expressed in inner ear then the gene is considered a candidate. Alternatively, the interacting genes from a specific locus list were compared against lists from other loci to identify if a hit was scored against proteins among two or more lists. The original genes corresponding to such interactor(s) were considered as candidates for the respective deafness loci. The strong candidates, based on the above analyses, for various deafness loci are presented in Table 5.

In principle, the interactions-based strategy can be targeted to identify candidates for deafness if a database for interacting proteins involved in inner ear development and function is available. For example, oncomodulin and prestin are expressed in outer hair cells [20]. The protein interaction approach could link the possible candidate genes to specific cochlear cells by identifying known interactants. If the interactors happen to map to a region harboring a deafness gene, such interactors are obvious candidates for mutational analysis. However, such an approach will require identification of interacting proteins. The primary limitation of the *in silico *approach described here is inadequate description of interacting protein networks.

The strong candidate list includes genes such as various cadherins, collagens, some cytoskeletal components and a number of growth factors and inner ear specific transcripts. For example, HAT (Human airway trypsin-like protease) from the DFNA27 locus is known to enhance cell growth and IL-8 production. It has been implicated in induction of PAR-2 (protease activated receptor)-mediated IL-8 release in psoriasis vulgaris [21]. Because HAT is expressed in the ear, and protease activated receptor (PAR-2) has the ability to activate G-proteins followed by an increase in calcium ion concentration, we consider HAT as a candidate. KDR(kinase insert domain receptor), a vascular endothelial growth factor(VEGF) receptor-type 2, from the same locus shows age-dependent expression in the inner ear [22]. Our analyses indicated that only a fraction (200/2400) of genes mapping to various genomic intervals was expressed in the inner ear. We attribute these observations to depth of inner ear libraries. It is likely that the genes being scored in these libraries have multiplicity for certain transcripts and absence of other transcripts. For example, out of 153 genes at the DFNA7 locus, only 18 genes are present in the cochlear library. We cannot reasonably rule out the expression of the remaining 135 genes in the inner ear. Therefore, the approach presented here will be more comprehensive if we do not include ear expression in this scheme. Consequently, in a second attempt to mine the protein-interaction data obtained from the HRPD, we analyzed all genes encoded in the candidate intervals for their interactors. The interaction data were considerably exhaustive and resulted in many more possible candidates with their expression not reported in the ear expression library. A summary of gene numbers at different loci before and after interacting proteins analysis using the ear-expression scheme is presented in Table 6. The mouse syntenic genes are also indicated in these results. The number of unfiltered candidate genes for each locus obtained by interacting proteins analysis is shown in Table 7. To elucidate the relevance of genes not found in the ear-expression library as possible candidates, we performed a literature search cross-referencing the identified gene with any reported hearing-associated condition in humans or other model animals. Some of these genes were linked to ear-development or hearing impairment as a secondary or unrelated symptom of other conditions. For example, Neurod1 gene mapping to DFNB27 locus was not reported in any of the inner ear libraries. However, it appears to participate in the development of the auditory system as NeuroD1 null mice exhibited severe reduction of sensory neurons in the cochlear-vestibular ganglion [23]. E2F3, a transcription factor of the E2F family mapping to the DFNA21 locus, may be indirectly implicated by its ability to regulate cell proliferation possibly during the developmental stages [24]. Other candidate genes from the unfiltered candidate analysis for the various loci are listed in Table 8. Thus the unfiltered strategy adds 51 candidates for 25 loci and expands the candidate list to 92 genes for further mutation analysis.

Our approach indicated the presence of possible candidates within most of the mapped loci. However, prediction of candidate genes was not easy for loci indicated by asterisks in Table 7, because the genes mapping to these loci did not fulfil the criteria we have employed. We further examined these genes on the basis of their reported function. The following description pertains to specific genes that are not indicated in the candidate lists. Within the DFNA16 locus, SCNA3 and SCNA2, both being voltage-gated sodium channels, can be considered candidates based on involvement of related sodium channels in hearing [25]. Similarly, ATP2C1 in DFNA18 locus is a likely candidate because mutations in a related ATPase have been shown in mice that are profoundly deaf and have a balance defect [26]. The EphB1 gene, within the DFNA18 locus, plays a major role during the development of the inner ear in mice [27]. The DFNA23 locus has six1 gene that plays a pivotal role in the control of the mouse otic vesicle patterning [28]. Neugrin, mapping to DFNA30 locus, appeared to be an appropriate candidate as it was shown to be up-regulated throughout neuronal differentiation [29]. A possible candidate for the DFNA47 locus is the transcription factor Nfib, an essential player in the maturation of lungs and brain development [30]. The splicing regulation carried out by Pnn, mapping to the DFNB5 region, is a reasonable candidate [31]. We believe the genes presented in this article may serve as starting candidates toward identifying molecular mechanisms for specific deafness phenotypes.

## Conclusion

We have used an *in silico *strategy to assemble a list of candidate genes that are positionally linked to and could be causing specific nonsyndromic hereditary hearing loss conditions. As presented here, a list of 2378 genes mapping to various genomic intervals have been narrowed down to 92 genes as candidates. These candidates may be analyzed for mutations in various deafness phenotypes in parallel with attempts to further narrow down the suspected region by genetic linkage analysis. It warrants mention that the potential of the approach presented here will be better harnessed as more information becomes available about inner ear transcripts and protein interaction networks.

## Methods

### Generating list of loci for *in silico *prediction

The list of most current information and identified loci for the various nonsyndromic hearing loss and syndromic forms was obtained from the Hereditary Hearing Loss Homepage and the survey of latest literature [4]. The list of deafness loci with unknown specific genes for the autosomal dominant, autosomal recessive, and syndromic forms was also compiled from the same web based source.

### GeneRetriever for EST identification within each deafness locus

A list of all cloned and identified genes from within each of the listed genomic intervals was obtained using GeneRetriever^®^, a Perl-based data mining software that has a simple graphical user interfaces [12]. It automatically retrieves from either NCBI or Ensembl databases information that includes all genes and transcripts located in a genomic interval flanked by two genetic markers.

### Database analysis

The list of genes and transcripts for each specific locus obtained using GeneRetriever^® ^was compared against two sets of ear gene-expression databases. The first set includes genes expressed in the developing ear [13]. This list is a compilation of the numerous genes that are expressed at different stages during inner ear development in two animal species. The second set was obtained from fetal cochlear cDNA library and EST database (updated as of 2002) of the Morton Hearing Research Group [14]. The data present in this set was adapted from Unigene [12]. The database has 14,805 ESTs, and 12,624 ESTs are sorted by Unigene into 4,519 independent clusters. Unigene did not classify the remaining ESTs due to factors such as possible contaminating sequences, very small inserts, or excessive repetitive elements. For a gene within a particular locus to be considered for candidacy, it has to be present in either of the above two databases. Genes that were not present in either expression databases were initially eliminated from consideration. It warrants mention that functional significance of expressed sequences in human and mouse inner ear has been used to propose deafness candidates [32,33].

### Human reference protein database

In comparing the two sets of databases to the list of genes and transcripts within each hereditary hearing loss locus obtained using GeneRetriever^®^, we were able to compile a list of possible candidate genes for the various loci. To further narrow-down and refine this list, we obtained a list of all known interacting genes for each of the known and candidate genes using the Human Reference Protein Database (HRPD)[15]. The interacting proteins for all the genes within the mapped loci were obtained regardless of whether the gene is present in the two data sets of inner-ear expressed transcripts. In our first attempt of mining the data, if a gene is not present in the data set but its interacting proteins are expressed or present in the cochlea or identified in the table of gene expression in the developing ear, then this gene is considered a candidate. In our second attempt, we removed the ear-expression filter requirement. Therefore, any interacting and repeating protein was given consideration. Identifying candidate interacting genes that repeat in many loci supported their candidacy, resulting in a more comprehensive candidate list.

## Authors' contributions

RPK was responsible for conceiving this project, designing the strategy, preparation of the manuscript and overall supervision. RA wrote some basic programs for comparisons, carried out data base mining, assembled the set of candidate genes and participated in manuscript preparation. CJT was involved in the initial stages of the project. All authors read and approved the final manuscript.

## Figures and Tables

**Table 1:** Autosomal dominant nonsyndromic loci.

Locus Name	Location
DFNA7	1q21–q23
DFNA16	2q24
DFNA18	3q22
DFNA21	6p21
DFNA23	14q21–q22
DFNA24	4q
DFNA25	12q21–24
DFNA27	4q12
DFNA30	15q25–26
DFNA31	6p21.3
DFNA32	11p15
DFNA34	1q44
DFNA37	1p21
DFNA41	12q24–qter
DFNA42	5q31.1–32
DFNA43	2p12
DFNA44	3q28–29
DFNA47	9p21–22
DFNA49	1q21–q23
DFNA50	7q32
DFNA53	14q11–q12
DFNA54	5q31

**Table 2:** Autosomal recessive nonsyndromic loci.

Locus Name	Location
DFNB5	14q12
DFNB13	7q34–36
DFNB14	7q31
DFNB15	3q21–q25, 19p13
DFNB17	7q31
DFNB19	18p11
DFNB20	11q25–qter
DFNB24	11q23
DFNB25	4p15.3–q12
DFNB26	4q31
DFNB27	2q23–q31
DFNB28	22q13
DFNB32	1p13.3–22.1
DFNB33	9q34.3
DFNB35	14q24.1–24.3
DFNB38	6q26–q27
DFNB39	7q11.22–q21.12
DFNB40	22q
DFNB42	3q13.31–q22.3
DFNB44	7p14.1–q11.22
DFNB46	18p11.32–p11.31
DFNB48	15q23–q25.1
DFNB49	5q12.3–q14.1.
DFNB55	4q12–q13.2
DFNB58	2q14.1–q21.2
DFNB60	5q22–q31

**Table 3:** GeneRetriever list of known genes found within the DFNA27 locus.

Gene ID	Gene Entrez	Type	GeneDescription	Expressed in Cochlear Library	Interactor Cochlear Protein
KDR	3791	Known	kinase insert domain receptor (a type III receptor tyrosine kinase)	Yes	VEGF A, Grb2, CDH5
FLJ13352	79644	Known	hypothetical protein FLJ13352	No	
TPARL	55858	Known	TPA regulated locus	Yes	
CLOCK	9575	Known	clock homolog (mouse)	No	
PDCL2	132954	Known	phosducin-like 2	No	
NMU	10874	Known	neuromedin U	Yes	
SEC3L1	55763	Known	SEC3-like 1 (S. cerevisiae)	No	
KIAA0635	9662	Known	KIAA0635	Yes	
KIAA1211	57482	Known	KIAA1211 protein	No	
MRPL22P1	359738	Known	mitochondrial ribosomal protein L22 pseudogene 1	No	
NRPS998	132949	Known	2-aminoadipic 6-semialdehyde dehydrogenase	No	
PPAT	5471	Known	phosphoribosyl pyrophosphate amidotransferase	Yes	
PAICS	10606	Known	phosphoribosylaminoimidazole carboxylase, succinocarboxamide synthetase	No	
SRP72	6731	Known	signal recognition particle 72kDa	No*	Caspase 3
ARL9	132946	Known	ADP-ribosylation factor-like 9	No	
GLDCP	2732	Known	glycine dehydrogenase (decarboxylase) pseudogene	No	
HOP	84525	Known	homeodomain-only protein	Yes	HDAC2
SPINK2	6691	Known	serine protease inhibitor, Kazal type 2 (acrosin-trypsin inhibitor)	No	
REST	5978	Known	RE1-silencing transcription factor	Yes	
C4orf14	84273	Known	chromosome 4 open reading frame 14	No	
POLR2B	5431	Known	polymerase (RNA) II (DNA directed) polypeptide B, 140kDa	Yes	
IGFBP7	3490	Known	insulin-like growth factor binding protein 7	No*	VEGF A, IGF1
SRIL	6644	Known	sorcin-like	No	
LPHN3	23284	Known	latrophilin 3	No	
EPHA5	2044	Known	EphA5	No	
CENPC1	1060	Known	centromere protein C 1	No	
BRDG1	26228	Known	BCR downstream signaling 1	No*	KIT
FLJ10808	55236	Known	hypothetical protein FLJ10808	Yes	
GNRHR	2798	Known	gonadotropin-releasing hormone receptor	No	
HAT	9407	Known	airway trypsin-like protease	Yes	PAR-2
FLJ16046	389208	Known	FLJ16046 protein	No	
YT521	91746	Known	splicing factor YT521-B	No*	KHDRBS3, FYN
DESC1	28983	Known	DESC1 protein	No	
UGT2B17	7367	Known	UDP glycosyltransferase 2 family, polypeptide B17	No	

*These genes are not listed in the human cochlear database. However, their interactors are present in the cochlear database.

**Table 4:** Cochlear-expressed EST found within DFNA27 locus.

Locus	Location	Genes from Cochlea	Genes from Mouse	Genes from Known Disorders
DFNA27	4q12	FLJ10808	EPHA5	None
		HAT	HAT	
		HOP	KDR	
		KIAA0635		
		KDR		
		NMU		
		POLR2B		
		PPAT		
		REST		
		TPARL		

**Table 5:** List of candidates for various deafness loci.

Loci	Location	Candidates	Loci	Location	Candidates
DFNA7	1q21–q23	ATP1B1	DFNB5	14q12	**
		F5	DFNB13	7q34–36	SLC37A3
		MYOC	DFNB17	7q31	WNT2
		SLC19A2	DFNB19	18p11	LAMA1
		POU2F1	DFNB20	11q25–qter	KCNJ1
DFNA16	2q24	*			TECTA
DFNA18	3q22	**			SLC37A2
DFNA21	6p21	*	DFNB27	2q23–q31	ITGA6
DFNA23	14q21–q22	**			SP3
DFNA25	12q21–24	HAL	DFNB28	22q13	KCNJ4
		SLC25A3			MT
		IGF1			SOX10
DFNA27	4q12	HAT	DFNB32	1p13.3–22.1	COL11A1
		KDR/VEGFR2			DR1
DFNA30	15q25–26	**			F3
DFNA31	6p21.3	TNF	DFNB33	9q34.3	TUBB2
		POU5F1			SLC34A3
DFNA34	1q44	**	DFNB35	14q24.1–24.3	NUMB
DFNA42	5q31.1	FGF1			FOS
		GFRA3	DFNB38	6q26–q27	QK1
		IK	DFNB39	7q11.22–q21.12	POR
		PCDH1	DFNB40	22q	CRYBB2
		DIAPH1			SLC25A1
		POU4F3			TBX1
DFNA47	9p21–22	**			

*The chromosomal regions for DFNA16 and DFNA21 code for 7 and 9 genes, respectively. However, none of these genes were listed in the mouse or human inner ear databases. The candidates as described in the text are based on their functional significance.

**The chromosomal regions for DFNA18, DFNA23, DFNA30, DFNA34, DFNA47 and DFNB5 code for a substantial number of genes. A small fraction of these genes are listed in human and mouse inner ear databases as shown in Table 6. Furthermore, no hits were scored by the protein interaction approach. Therefore the genes scoring hits in the ear databases may be considered as candidates and prioritized based on their function. Some of these priority candidates are described in the text.

**Table 6:** Summary of gene numbers from expression-library filtered analysis.

Condition	Map Location	Interval	Number of Genes	Mouse Synteny/Chromo #	Mouse Genes	Human Cochlear Genes	Mouse Inner ear Genes	Interacting Proteins	Shared Interactors
DFNA7	1q21–q23	18.6 Mb	153	1	161	18	6	15	5
DFNA16	2q24	2.6 Mb	7	2	16	0	0	0	0
DFNA18	3q22	12 Mb	116	6, 9	111	18	4	9	1
DFNA21	6p21	3.5 Mb	9	10	48	0	0	0	0
DFNA23	14q21–q22	8 Mb	79	12	60	7	1	4	0
DFNA25	12q21–24	14 Mb	108	10, 5	102	17	2	11	3
DFNA27	4q12	15 Mb	71	5	97	10	3	7	2
DFNA30	15q25–26	7 Mb	74	7	58	6	1	2	0
DFNA31	6p21.3	7.5 Mb	304	13, 17	385	13	1	8	2
DFNA34	1q44	5 Mb	88	1	23	3	0	1	0
DFNA42	5q31	12 Mb	176	13, 18	153	21	5	16	6
DFNA47	9p21–22	9 Mb	65	4	79	6	1	3	0
DFNB5	14q12	5.5 Mb	18	12	37	2	0	2	0
DFNB13	7q34–36	1.4 Mb	14	6	15	1	0	2	1
DFNB17	7q31	6.5 Mb	26	6	43	3	1	2	1
DFNB19	18p11	2.8 Mb	13	17	11	1	1	1	1
DFNB20	11q25–qter	13.4 Mb	152	9	263	14	1	6	3
DFNB27	2q23–q31	11 Mb	79	2	95	14	3	3	2
DFNB28	22q13	6.5 Mb	146	15	154	15	5	7	3
DFNB32	1p13.3–22.1	16 Mb	74	3, 5	89	13	3	3	3
DFNB33	9q34.3	3 Mb	86	2	54	3	4	2	2
DFNB35	14q24.1–24.3	8.2 Mb	116	12	103	8	4	3	2
DFNB38	6q26–q27	3.4 Mb	5	17	3	1	0	1	1
DFNB39	7q11.22–q21.12	18 Mb	114	5	108	10	2	2	0
DFNB40	22q11.21–12.1	9 Mb	285	5, 10, 16	76	10	4	4	3

Total			2378		2344	214	52	114	41

**Table 7:** Summary of gene numbers from unfiltered analysis.

Condition	Map Location	Interval	Number of Genes	Interacting Proteins
DFNA7	1q21–q23	18.6 Mb	153	29
DFNA16	2q24	2.6 Mb	7	1
DFNA18	3q22	12 Mb	116	21
DFNA21	6p21	3.5 Mb	9	3
DFNA23	14q21–q22	8 Mb	79	29
DFNA25	12q21–24	14 Mb	108	33
DFNA27	4q12	15 Mb	71	9
DFNA30	15q25–26	7 Mb	74	18
DFNA31	6p21.3	7.5 Mb	304	48
DFNA34	1q44	5 Mb	88	19
DFNA42	5q31	12 Mb	176	48
DFNA47	9p21–22	9 Mb	65	19
DFNB5	14q12	5.5 Mb	18	5
DFNB13	7q34–36	1.4 Mb	14	6
DFNB17	7q31	6.5 Mb	26	9
DFNB19	18p11	2.8 Mb	13	3
DFNB20	11q25–qter	13.4 Mb	152	24
DFNB27	2q23–q31	11 Mb	79	12
DFNB28	22q13	6.5 Mb	146	27
DFNB32	1p13.3–22.1	16 Mb	74	11
DFNB33	9q34.3	3 Mb	86	8
DFNB35	14q24.1–24.3	8.2 Mb	116	19
DFNB38	6q26–q27	3.4 Mb	5	1
DFNB39	7q11.22–q21.12	18 Mb	114	12
DFNB40	22q11.21–12.1	9 Mb	285	25

Total			2378	439

**Table 8:** Candidate genes from unfiltered HPRD analysis*.

DFNB13	RAB19B	DFNA7	CREG
DFNB17	CAV2		DPT
	CAPZA2	DFNA18	PLXND1
	KCND2	DFNA21	E2F3
	ING3	DFNA23	DAAM1
DFNB19	PTPRM		MNAT1
DFNB20	GRIK4	DFNA25	HSyn
	NRGN		SELPLG
	RICS	DFNA27	SRP72
DFNB27	MTX2		IGFBP7
	TTN		BRDG1
	NEUROD1		YT521
DFNB28	RANGAP1	DFNA30	CIB1
DFNB32	NTNG1		PRC1
DFNB33	TRAF2	DFNA31	ABT1
	NPDC1		UBD
DFNB35	PGF	DFNA34	SMYD3
	NGB		CIAS1
DFNB39	UPK3B	DFNA42	NEUROG1
	PCLO		TTID
	GRM3		NRG2
DFNB40	CLTCL1		PCDHAC1
	TXNRD2		PCDHAC2
	SDF2L1		NDFIP1
	MMP11	DFNA47	CDKN2A
	CABIN1		

*The products of these genes shared interacting proteins with genes mapping to other deafness intervals. The above genes may be combined with the candidates predicted by the functional analysis and listed in Table 5.
